# Vegetation-Driven Changes in Soil Salinity Ions and Microbial Communities Across Tidal Flat Reclamation

**DOI:** 10.3390/microorganisms13061184

**Published:** 2025-05-22

**Authors:** Shumei Cai, Sixin Xu, Deshan Zhang, Yun Liang, Haitao Zhu

**Affiliations:** 1 Institute of Eco-Environment and Plant Protection, Shanghai Academy of Agricultural Sciences, Shanghai 201403, China; caishumei@saas.sh.cn (S.C.);; 2 Key Laboratory of Low-Carbon Green Agriculture, Ministry of Agriculture and Rural Affairs, Shanghai 201403, China; 3 Shanghai Key Laboratory of Horticultural Technology, Shanghai 201403, China

**Keywords:** tidal flat, salinity ions, soil bacterial community, reclamation stage, vegetation cover type

## Abstract

Soil microbes play a vital role in tidal flat ecosystems but are highly susceptible to disturbances from land reclamation. This study investigated the dynamics of bacterial communities and their environmental drivers across a 50-year reclamation chronosequence under three vegetation types (bare flats, reed beds, and rice fields). The results showed that, after 50 years of reclamation, total dissolved salts decreased significantly in vegetated zones, particularly in rice fields, where Cl^−^ dropped by 54.71% and nutrients (SOC, TN, TP) increased substantially. Key ions, including HCO_3_^−^, Cl^−^, and K^+^, were the primary drivers of microbial community structure, exerting more influence than total salinity (TDS) or pH. Bacterial abundance and diversity increased over time, with rice fields showing the highest values after 50 years. Actinobacteriota and Proteobacteria were positively correlated with HCO_3_^−^ and K^+^, while Cl^−^ negatively affected Acidobacteriota. Genus-level analyses revealed that specific taxa, such as *Sphingomonas* and *Gaiella*, exhibited ion responses diverging from broader phylum-level patterns, exemplifying niche-specific adaptations to salinity regimes. These findings underscore the pivotal role of vegetation type and individual salinity ions in driving microbial succession during tidal flat reclamation. A phased vegetation strategy, starting with reed colonization and followed by rice cultivation, can enhance soil quality and microbial diversity. This research provides important insights for optimizing vegetation management and ion monitoring in sustainable tidal flat reclamation.

## 1. Introduction

Tidal flats in estuarine and coastal regions are invaluable natural assets that support a wide range of ecosystem functions critical for both environmental stability and sustainable land use. These transitional zones serve as biodiversity reservoirs and carbon sinks, while facilitating active biogeochemical cycling through sediment–water exchanges, organic matter deposition, and microbial activity. Their ecological functions are closely regulated by dynamic interactions among vegetation, soil properties, and microbial communities, which jointly modulate nutrient availability, salinity gradients, and elemental fluxes across the land–sea interface [1]. However, over the past three decades, global tidal flat areas have declined by 16%, primarily due to coastal development, sea-level rise, and reduced sediment supply [2,3,4]. Anthropogenic reclamation, which converts tidal flats into agricultural or urban land, has profoundly disrupted these ecosystems by altering hydrology, sediment dynamics, and vegetation succession [5,6]. Therefore, investigating biodiversity changes in reclaimed tidal flats is crucial for understanding their ecological trajectory and guiding sustainable management strategies.

Soil microbial communities in tidal flats act not merely as passive responders but as active regulators of ecosystem function, deeply involved in key biogeochemical processes that influence vegetation succession, soil salinity ion distribution, and overall ecological trajectories [7]. Microbial communities regulate key biogeochemical cycles by modulating redox conditions, pH, and the availability and distribution of major salinity-related ions such as Na^+^, K^+^, Cl^−^, HCO_3_^−^, and SO_4_^2^^−^. These microbial processes actively reshape the soil ionic environment, influencing osmotic balance and nutrient dynamics, which in turn affect plant colonization and successional trajectories. As vegetation develops, microbial diversity and functional complexity increase, reinforcing ecological stability and enhancing plant–soil feedbacks. Acting as ecosystem engineers, tidal flat microbial communities play a central role in shaping soil properties and vegetation patterns. Understanding these microbial-driven mechanisms is essential for predicting and managing ecosystem responses in coastal zones undergoing anthropogenic reclamation and environmental change.

During tidal flat reclamation, vegetation typically follows a successional trajectory, from unvegetated bare flats to natural colonization by halophytic species (e.g., *Phragmites australis*, *Suaeda salsa*) and eventually to the cultivation of salt-tolerant crops such as rice (*Oryza sativa*) [8]. Each stage of vegetation development imposes distinct modifications on the soil environment, thereby influencing microbial community assembly [9]. Bare flats are often characterized by high salinity, poor aeration, and low organic matter, supporting a taxonomically limited and stress-tolerant microbial assemblage. As halophytes establish, their root systems enhance rhizosphere oxygenation and exude organic substrates, improving redox conditions and promoting the diversity and activity of heterotrophic taxa [10,11]. Subsequently, rice cultivation under flooded and nutrient-rich conditions introduces strong anthropogenic influences, leading to shifts toward heterotrophic and anaerobic microbial taxa. These vegetation-driven changes reshape bulk soil properties and modulate salinity-related ion profiles, which remain crucial but insufficiently explored across the vegetation succession gradients.

Saline soils challenge microbial and vegetation survival through osmotic stress, ionic toxicity, and metabolic inhibition. While previous studies have primarily focused on total salinity as the main stressor, recent research highlights the ion specificity of microbial responses [12,13]. Sodium (Na^+^) disrupts enzyme function and membrane integrity, while potassium (K^+^) serves as a compatible solute, stabilizing cellular osmotic balance [14]. Chloride (Cl^−^) inhibits nitrogenase activity in diazotrophs, whereas sulfate (SO_4_^2−^) supports sulfur-oxidizing bacteria but suppresses methanogens [15,16,17]. Bicarbonate (HCO_3_^−^) and carbonate (CO_3_^2−^) further influence pH-dependent processes, such as ammonia volatilization and phosphorus solubilization [18]. These ion-specific interactions create complex selection pressures that are often obscured by bulk salinity measurements. For instance, high Na^+^/K^+^ ratios impair microbial osmoregulation even under moderate total salinity, while elevated HCO_3_^−^ can mitigate aluminum toxicity in alkaline soils [14]. Understanding the distinct roles of individual ions is essential for enhancing microbial diversity, nutrient cycling, and plant colonization in tidal flats, thereby supporting the stability and resilience of coastal ecosystems.

The aim of this study is to elucidate how tidal flat reclamation duration and vegetation cover regulate saline-ion-driven microbial community assembly. More specifically, the objectives are to investigate whether dominant salinity ion composition rather than total salinity governs bacterial diversity patterns and whether vegetation-mediated soil amelioration processes differentially shape microbial niche partitioning across reclamation stages. To achieve this, we compare soil bacterial community composition, α-diversity, relative abundance, and ionic profiles across three vegetation types (bare flats, reed beds, and rice fields) along a 50-year reclamation chronosequence. We hypothesize that tidal flats with long-term vegetation establishment will host more diverse microbial communities dominated by ionic-stress-tolerant taxa and that specific ions will serve as predictive biomarkers for microbial community succession during tidal flat reclamation.

## 2. Materials and Methods

### 2.1. Study Site

The study site is located on Hengsha Island, Shanghai (N 31°16′–31°23′, E 121°47′–122°9′), and is characterized by a subtropical monsoon climate, with an average annual temperature of 15.4 °C. With an average annual rainfall of 1100 mm and 2200 h of sunshine annually, the island, formed by sediment deposition from the Yangtze River at the eastern end of its estuary, covers a total area of 60 km^2^, including 26.8 km^2^ of arable land. Historically, due to persistent wind and wave erosion along with tidal action, the island has undergone a southeast subsidence evolution pattern characterized by a northwestward rising trend, gradually migrating northwestward, albeit with a net increase in overall area. Since the implementation of the sea pond reinforcement and shore protection project in 1958, erosion along the island’s southern coast has been mitigated, resulting in stabilized shorelines and tidal flats.

### 2.2. Soil Sampling Plot

**Figure 1:**
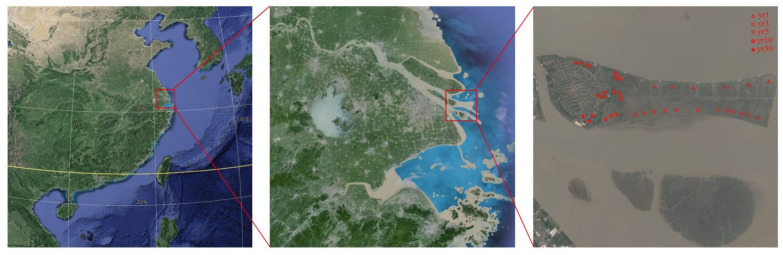
Geographic locations of the study area and sampling sites. yr1, yr3, yr5, yr10, and yr50 denote 1, 3, 5, 10, and 50 years after reclamation, respectively.

Coastal tidal flat soil sampling sites were selected in areas where seabed sediment deposition and subsequent soil matrix formation occurred following dike construction. These sampling sites exhibited minimal profile differentiation while retaining the granulometric characteristics of the original blowing mat. The mean particle size distribution at the sampling sites comprised 28.6% sand (>63 μm), 50.3% silt (4–63 μm), and 21.0% clay (<4 μm), classified according to the Wentworth sediment classification system [19]. Based on the duration of land reclamation and the succession of typical vegetation types, five representative reclamation chronosequences were identified in the study area, corresponding to approximately 1, 3, 5, 10, and 50 years after reclamation. These five stages were further grouped into three major reclamation phases. The early phase (1, 3, and 5 years) was characterized by the presence of two dominant vegetation types: bare beach and reed beds. In contrast, the middle (10 years) and late (50 years) phases included three vegetation types: bare tidal flat, reed bed, and rice field. These stages represent distinct land use patterns and ecological conditions under the combined influence of natural succession and anthropogenic activities. To ensure the ecological and spatial representativeness of the sampling design, plot selection followed three key criteria: (1) the vegetation types were widely distributed and ecologically representative of their respective reclamation stages; (2) three replicate plots (20 m × 20 m) were established for each vegetation type, with a minimum spacing of 1 km between plots to reduce spatial autocorrelation; and (3) the plots were evenly distributed across the study area to capture spatial environmental heterogeneity. Furthermore, site selection was supported by a combination of high-resolution satellite imagery, historical land use records, and detailed field reconnaissance. In total, 36 plots were established as illustrated in Figure 1.

### 2.3. Determination of Soil Physiochemical Properties

In August 2022, surface soil samples (0–20 cm depth) were collected from each plot. For each plot, ten subsamples were taken using the S-shaped sampling method and then thoroughly mixed to obtain a composite sample. Approximately 1.0 kg of soil was collected for each composite sample. The mixed soil samples were passed through a 4 mm sieve to remove large debris and homogenize the material. Each sample was then divided into two portions for subsequent analyses: (1) approximately 500.0 g of soil was air-dried for the determination of basic physicochemical properties, and (2) approximately 10.0 g of soil was stored at −20 °C for high-throughput sequencing and real-time quantitative polymerase chain reaction (qPCR) analysis.

Soil physiochemical properties were analyzed following standard procedures described by Lu [20], with specific methods as follows: Soil organic carbon (SOC) was determined using the potassium dichromate oxidation method with external heating. Total nitrogen (TN) was measured via the Kjeldahl digestion–distillation method. Total phosphorus (TP) and total potassium (TK) were analyzed after HF–HClO_4_ acid digestion, with TP determined colorimetrically using the molybdenum blue method and TK via flame atomic absorption spectrophotometry. Soil pH was measured in a 1:2.5 (soil:water, *w*/*v*) suspension using a calibrated pH meter (FE28, Mettler Toledo, Giessen, Germany) after a 30 min equilibration. Total dissolved solids (TDS) were determined gravimetrically by evaporating a known volume of soil–water extract at 105 °C. Exchangeable cations including Ca^2^^+^, Mg^2^^+^, Na^+^, and K^+^ were extracted with 1 mol·L^−^^1^ ammonium acetate (pH 7.0) and measured using atomic absorption spectrophotometry. Carbonate (CO_3_^2−^) and bicarbonate (HCO_3_^−^) were quantified by acid-neutralization titration using phenolphthalein and methyl orange as indicators. Chloride (Cl^−^) was measured via silver nitrate titration and sulfate (SO_4_^2−^) via the turbidimetric method with spectrophotometric detection. 

### 2.4. Soil DNA Extraction and Bacterial Community Analysis

For high-throughput sequencing analysis, 0.5 g of each soil sample was used for soil DNA extraction using the MoBio PowerSoil^®^ DNA Kit (Tiangen Biotech Co., Ltd., Beijing, China) according to the manufacturer’s instructions. The quality and concentration of DNA were determined using 1.0% agarose gel electrophoresis and a NanoDrop^®^ ND-2000 spectrophotometer (Thermo Fisher Scientific Inc., Waltham, MA, USA) and it was kept at −80 °C prior to further use. Purified amplicons were pooled in equimolar amounts and paired-end-sequenced on an Illumina MiSeq PE300 platform (Illumina, San Diego, CA, USA). The V3–V4 region of the 16 S rRNA gene was amplified from the isolated bacterial DNA via the primer set 338 F/806 R (5′-ACTCCTACGGGAGGCAGCAG-3′ and 5′-GGACTACHVGGGTWTCTAAT-3′) in an ABI GeneAmp 9700 PCR system (Applied Biosystems, Foster City, CA, USA). The PCR mixture included 4 μL of 5 × Fast Pfu buffer, 2 μL of 2.5 mM dNTPs, 0.8 μL of each primer (5 μM), 0.4 μL of Fast Pfu polymerase, 10 ng of template DNA, and ddH_2_O to a final volume of 20 µL. The PCR amplification cycling conditions were as follows: an initial denaturation at 95 °C for 3 min, followed by 27 cycles of denaturation at 95 °C for 30 s, annealing at 55 °C for 30 s and extension at 72 °C for 45 s, and a single extension at 72 °C for 10 min. All samples were amplified in triplicate. The PCR product was extracted from a 2% agarose gel and purified via the AxyPrep DNA Gel Extraction Kit (Axygen Biosciences, Union City, CA, USA). The purified DNA was sent to Biozeron Biotechnology Co., Ltd. (Shanghai, China) for Illumina MiSeq sequencing.

### 2.5. qPCR Analysis

qPCR was used to examine the effects of the reclamation stage and vegetation type on soil bacterial abundance. Extracted DNA was examined using a NanoDrop spectrophotometer and visually checked on agarose gel (1% *w*/*v*) before qPCR quantification. Standard reactions were performed for all samples in triplicate with an ABI 7500 Real-time PCR System (Applied Biosystems, Foster City, CA, USA) via the SYBR green qPCR method. The qPCR mixture (25 μL) contained 12.5 μL of Maxima SYBR Green/ROX qPCR Master Mix (Fermentas, Vilnius, Lithuania), 1 μL of each primer (338 F/806 R, 5 μM), 5 μL of template DNA, and 5.5 μL of ddH_2_O. The specificity of qPCR amplification was confirmed by analyzing melting curves and performing gel electrophoresis. In all experiments, the same procedure was applied to negative controls without template DNA to eliminate contamination. Gene abundance in each reaction was calculated on the basis of the constructed standard curves and then converted to copies per gram of soil, assuming 100% DNA extraction efficiency.

### 2.6. Statistical Analysis

The amplicon sequence variants (ASVs) were obtained using the DADA2 algorithm in QIIME 2 (v2022.2). The raw sequencing reads were deposited into the NCBI Sequence Read Archive (SRA) database under the accession number SRP415876. Taxonomic annotation was conducted via the RDP and UNITES databases to identify bacterial taxa.

After assessing normality (Shapiro–Wilk test) and homogeneity of variance (Levene’s test), the data significantly deviated from parametric assumptions (normality: *p* < 0.05; homoscedasticity: *p* < 0.05; Table S1). To address these violations, non-parametric tests were selected for their robustness to non-normal distributions and heteroscedasticity. The effects of vegetation type and reclamation duration on soil physiochemical properties were analyzed using the Scheirer–Ray–Hare test, which extends the Kruskal–Wallis test to multifactorial designs without requiring normality or equal variances.

Bacterial abundance and diversity were quantified via the Shannon index, implemented in Mothur (v1.30.1). PCoA of the Bray–Curtis distance between the samples was used to characterize the similarity of bacterial communities among the treatments. The vegan data package within R (v4.3.2) was used for redundancy analysis (RDA) to identify factors that affected the bacterial community. Additionally, Spearman correlation analysis was used to explore the relationships between bacterial diversity and environmental factors.

## 3. Results

### 3.1. Changes in Soil Physicochemical Properties During Reclamation

To better understand the soil–microbe–vegetation interactions, we first analyzed temporal changes in soil physicochemical properties across different vegetation types during tidal flat reclamation (Table 1). In the early stages (years 1, 3, and 5), soil parameters such as SOC, TN, TP, TK, TDS, and pH fluctuated dynamically under both bare tidal flat and reed bed conditions, reflecting the transitional phase of soil development following initial reclamation.

In the later stages (year 10 and year 50), soil properties exhibited a more stabilized pattern. Soil pH remained relatively stable across vegetation types, while TK declined significantly. However, other soil parameters varied depending on vegetation type. In rice fields, SOC, TN, and TP increased markedly by year 50, accompanied by a pronounced decrease in TDS. In reed beds, TP and TDS declined significantly, while SOC and TN remained relatively unchanged. In bare flats, SOC, TN, and TP increased significantly, but TDS did not change appreciably. By year 50, rice fields showed the greatest enrichment in key nutrients: SOC, TN, and TP were 50.35%, 48.24%, and 30.96% higher than those in reed beds and 74.79%, 71.01%, and 34.91% higher than in bare flats, respectively. Concurrently, TDS declined by 21.31% in rice fields and 21.65% in reed beds relative to year 10, while pH remained stable.

### 3.2. Influence of Ionic Composition on Total Dissolved Salt and pH

To assess whether specific salinity ions exert greater influence on microbial communities than overall salinity levels, we further examined the ionic composition of the soil solution. The soils were identified as sulfate–chloride saline types, primarily composed of Cl^−^ and SO_4_^2−^, followed by HCO_3_^−^, while CO_3_^2−^ remained below detection limits. On average, Cl^−^, SO_4_^2−^, and HCO_3_^−^ accounted for 50.20%, 35.36%, and 32.35% of total anions, respectively (Table S2). The predominant cations were Ca^2^^+^ and Na^+^, with lower concentrations of Mg^2^^+^ and K^+^. Ion composition varied significantly across reclamation years, particularly during the initial 1–5 years. Over time, marked declines in Cl^−^ were observed in soils from rice fields and reed beds, with reductions of 54.71% and 37.42%, respectively, by year 50 compared to year 10. In contrast, Cl^−^ increased by 11.36% in bare flats.

**Figure 2:**
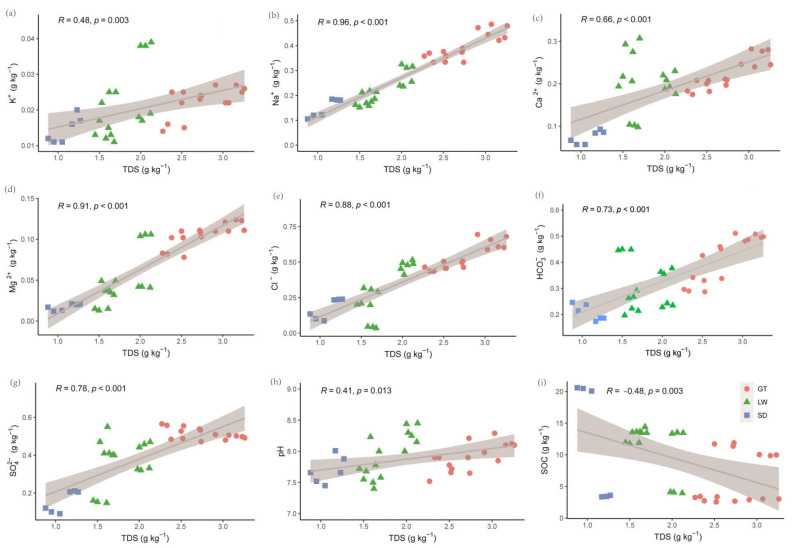
Variations in Pearson correlation coefficients among soil parameters: K^+^ (**a**), Na^+^ (**b**), Ca^2^^+^ (**c**), Mg^2^^+^ (**d**), Cl^−^ (**e**), HCO_3_^−^ (**f**), SO_4_^2−^ (**g**), pH (**h**), SOC (**i**), and TDS across different vegetation cover types. GT: bare flat; LW: reed bed; SD: rice field.

Correlation analysis revealed that TDS was strongly positively correlated with Na^+^ (R = 0.96), Mg^2^^+^ (R = 0.91), and Cl^−^ (R = 0.88), among others (*p* < 0.01), indicating that these ions are primary contributors to soil salinity (Figure 2). Additionally, TDS was significantly positively correlated with pH (R = 0.41, *p* = 0.013) and negatively correlated with SOC (R = −0.48, *p* = 0.003). Regarding pH, strong positive correlations were observed with K^+^ (R = 0.51), Mg^2^^+^, and Na^+^, while Cl^−^ also showed a significant positive correlation. No significant correlations were found between pH and HCO_3_^−^, SO_4_^2−^, or SOC (Figure S1). These findings indicate that specific ions, particularly Na^+^, Cl^−^, and K^+^, rather than TDS alone, play a pivotal role in shaping soil chemical properties relevant to microbial habitats.

### 3.3. Changes in Soil Bacterial Community Structure During Reclamation

**Figure 3:**
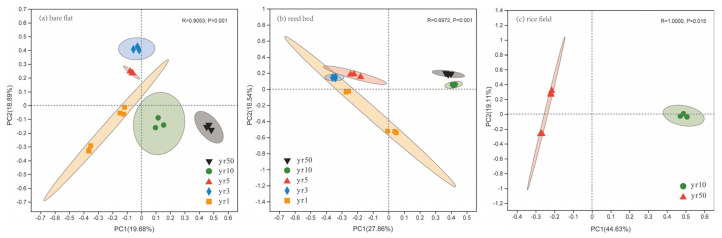
Principal coordinates analysis (PCoA) of soil bacterial communities at the ASV level across reclamation years (yr1, yr3, yr5, yr10, and yr50) in tidal flats. (**a**) Bare flat; (**b**) reed bed; (**c**) rice field.

To assess microbial shifts in response to reclamation and salinity ions, we analyzed 661,211 high-quality reads, yielding 35,753 amplicon sequence variants (ASVs) based on 97% sequence identity across 36 soil samples. Principal coordinate analysis (PCoA) based on Bray–Curtis distances revealed distinct differences in 16 S bacterial community composition across reclamation years under all vegetation types (Figure 3). Significant temporal shifts were observed in bare tidal flats and reed beds between years 1, 3, 5, 10, and 50 (*p* = 0.001) and in rice fields between years 10 and 50 (*p* = 0.015).

**Figure 4:**
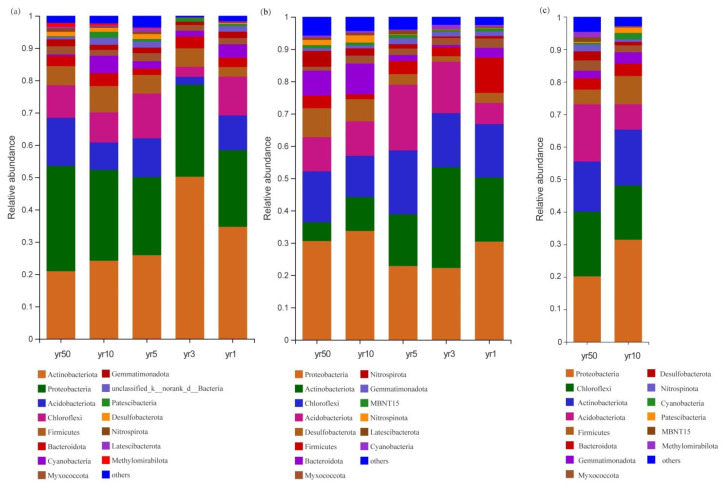
Community composition of soil bacterial phyla in tidal flats across different reclamation years (1, 3, 5, 10, and 50) and vegetation cover types. (**a**) Bare tidal flat; (**b**) Reed bed; (**c**) Rice field. Taxa with relative abundances < 0.01 are grouped as “others”.

Reclamation induced marked changes in bacterial phylum composition over 50 years (Figure 4). In reed beds, the relative abundance of Actinobacteria decreased from 34.68% to 20.93%, while Proteobacteria increased from 23.73% to 32.61%. Conversely, in rice fields, Proteobacteria declined from 31.47% to 20.20%, accompanied by an increase in Acidobacteria from 7.79% to 17.59%. These differential patterns suggest that prolonged land use changes selectively influence bacterial community structure through vegetation-specific modifications of soil physicochemical properties.

Tidal flat reclamation also induced distinct genus-level shifts in soil bacterial communities, with patterns strongly modulated by vegetation type and reclamation duration (Figure S2). In bare tidal flats (Figure S2a), dominant genera exhibited the highest temporal variability and inter-taxon divergence, with a marked structural shift occurring in year 3, suggesting rapid microbial niche turnover under saline stress in unvegetated soils. In contrast, reed beds (Figure S2b) showed more gradual changes in genus composition, with a major transition observed in year 5, potentially reflecting vegetation-induced shifts in soil properties and ionic composition. Rice fields (Figure S2c) were dominated by *norank_f__norank_o__Vicinamibacterales*, *norank_f__Vicinamibacteraceae*, and *norank_f__Anaerolineaceae*, all of which sharply increased across time, indicating selective enrichment of ion-adapted taxa. These patterns highlight the role of vegetation cover and soil development in modulating salinity-driven microbial succession.

### 3.4. Changes in Soil Bacterial Diversity and Abundance During Reclamation

**Figure 5:**
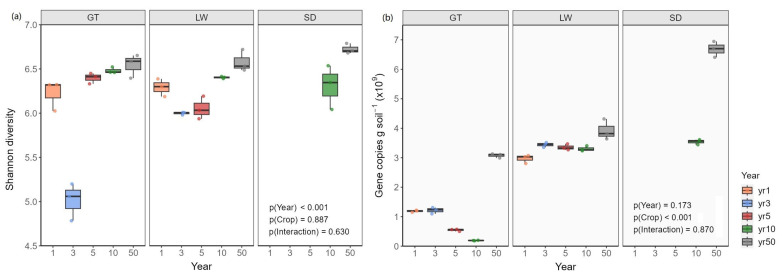
Variations in (**a**) bacterial Shannon diversity index and (**b**) gene copy numbers in tidal flat soils across different reclamation years (1, 3, 5, 10, and 50) and vegetation cover types. GT: bare tidal flat; LW: reed bed; SD: rice field.

Consistent with improved soil conditions over time, bacterial Shannon diversity increased significantly (*p* < 0.001) with reclamation progression across all vegetation types (Figure 5). The lowest diversity was observed in years 1 and 3, followed by a marked increase from year 5 onward. The highest diversity occurred in year 50, particularly in rice fields. Bacterial abundance, estimated via 16 S rRNA gene copy number, was also significantly higher in year 50 across all vegetation types (*p* < 0.001), reaching 6.68 × 10⁹ copies g^−^^1^ soil in rice fields. Compared with bare flats and reed beds, rice field soils exhibited 117.25% and 70.28% more gene copies, respectively, highlighting the strong influence of vegetation cover on microbial biomass.

**Figure 6:**
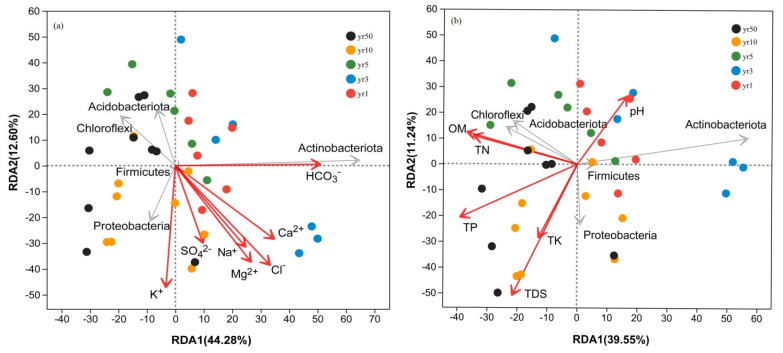
Redundancy analysis (RDA) of soil bacterial communities at the phylum level in relation to selected environmental variables. (**a**) Correlation between soil salinity ion composition and bacterial community structure. (**b**) Correlation between major soil chemical properties and bacterial community structure. Colored points represent samples from different reclamation years (1, 3, 5, 10, and 50). Grey arrows indicate species, while red arrows indicate environmental variables.

Redundancy analysis (RDA) confirmed that salinity ion composition, more than total nutrient levels or pH, was the primary driver of bacterial community structure (RDA1 = 44.28%, RDA2 = 12.60%) (Figure 6). Among the measured ions, HCO_3_^−^ (r^2^ = 0.615), Cl^−^ (r^2^ = 0.425), and K^+^ (r^2^ = 0.413) exhibited the strongest influences on soil microbial community structure (*p* < 0.01; Table S3), suggesting that specific ions play a dominant role in shaping microbial assemblages. Additionally, SOC, TN, and TP also contributed significantly, though to a lesser extent.

**Figure 7:**
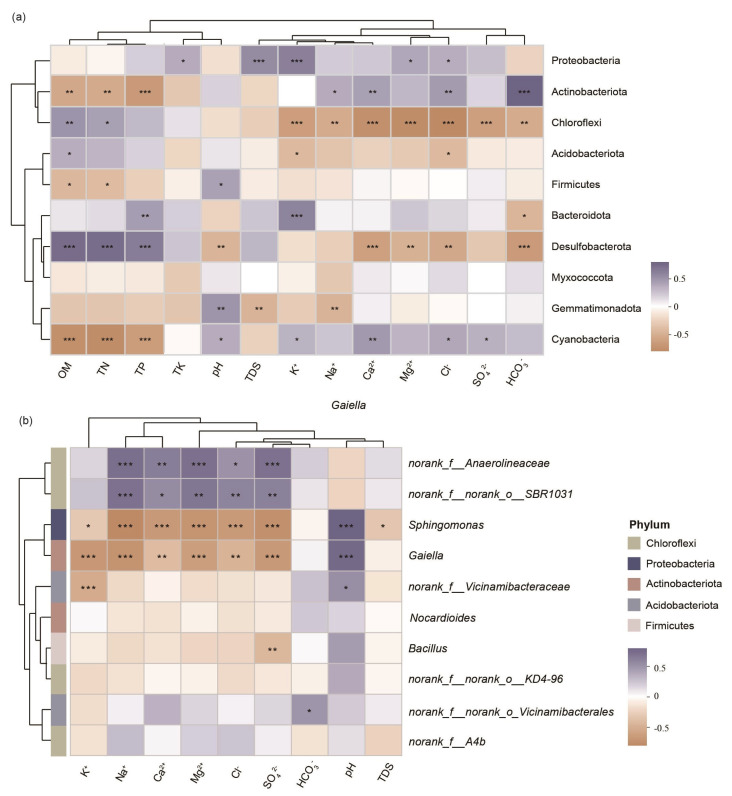
Spearman correlation heatmaps between environmental factors (*X*-axis) and the top ten bacterial taxa (*Y*-axis) at two taxonomic levels: (**a**) Phylum; (**b**) Genus. Color gradients represent correlation coefficients (R values), with red and blue indicating positive and negative correlations, respectively. Clustering dendrograms for taxa and environmental factors are shown on the left and top. Significance levels: * 0.01 < *p* < 0.05; ** 0.001 < *p* < 0.01; *** *p* < 0.001.

Taxonomically, Actinobacteriota showed a strong positive correlation with HCO_3_^−^ (*p* < 0.001) but significant negative associations with OM, TN, and TP (*p* < 0.01), while Proteobacteria were predominantly linked to K^+^ and TDS (*p* < 0.001), consistent with their osmoadaptive strategies (Figure 7a). In contrast, Chloroflexi exhibited consistent negative correlations with dominant salinity ions (*p* < 0.01), suggesting a generalized sensitivity to ionic stress. At the genus level (Figure 7b), *Sphingomonas* (Proteobacteria) and *Gaiella* (Actinobacteriota) showed strong antagonism toward most salinity ions, contrasting with their respective phylum-level trends. In parallel, unclassified taxa such as *norank_f_Anaerolineaceae* (Chloroflexi) and *norank_f_norank_o_SBR1031* (Chloroflexi) demonstrated unexpected positive correlations with salinity ions, indicating niche-specific adaptations diverging from their phylum’s overall salt-avoidance strategy.

## 4. Discussion

### 4.1. Tidal Flat Reclamation Shapes Soil Bacterial Community Assembly Across Vegetation Types and Time Scales

Our results show that tidal flat reclamation significantly restructures the soil bacterial community, influenced by both vegetation cover type and reclamation duration. These findings are consistent with those of previous studies [10,21] and are likely attributable to similar geographic distribution patterns [22] and prevalent soil types in silty coastal tidal flats [23]. The predominant phyla, Actinobacteria, Proteobacteria, Acidobacteria, and Chloroflexi, undergo notable compositional shifts (Figure 3), which have key implications for ecosystem functioning.

For example, in rice fields, the increased abundance of Chloroflexi and decreased abundance of Proteobacteria and Firmicutes with prolonged reclamation suggest a transition in microbial metabolic pathways, potentially reflecting changes in carbon fixation and nitrogen cycling capacity. Chloroflexi’s anaerobic nature and role in photosynthetic CO_2_ fixation may support more efficient organic matter turnover in saturated, reduced soil environments [24]. In contrast, the decline of nitrogen-cycling taxa such as Proteobacteria may indicate a reduction in denitrification potential [25], possibly due to reduced redox potential (Figure S3) and altered nitrogen availability [26]. These microbial shifts likely influence overall nutrient cycling and greenhouse gas fluxes [27], reflecting a coupling between microbial succession and soil function.

In reed beds and bare flats, the decline of Actinobacteria, key organic matter decomposers, suggests slower carbon turnover under saline and nutrient-poor conditions. The lower soil quality in reed beds and bare tidal flats compared to rice fields may be attributed to prolonged seawater soaking, resulting in higher soil salinity, greater bulk weight, and lower nutrient content. The observed rise in Desulfobacteriota in reed beds further highlights enhanced sulfur metabolism, likely mediated through root–microbial interactions and organic inputs from decaying reed biomass [28,29]. Such transitions may improve the resilience of halophytic vegetation to abiotic stress, contributing to soil stabilization and nutrient enrichment over time.

### 4.2. Salinity Ions as Primary Drivers of Microbial Community Restructuring

This study provides strong evidence that specific salinity ions, rather than total salinity, predominantly govern the structure of microbial communities during reclamation. In particular, HCO_3_^−^, Cl^−^, and K^+^ were most strongly correlated with changes in bacterial composition (Figure 6, Table S3). These ions appear to exert selective pressures that favor either salt-tolerant or salt-sensitive taxa. It has been widely reported that increasing salinity ions typically lead to a decrease in soil microbial biomass due to cell dehydration and lysis caused by osmotic stress [16]. However, salt-tolerant microorganisms counteract these challenges by accumulating osmolytes for adaptation. Notably, external factors can also impact microbial diversity and community formation to varying extents [5].

In our study, Actinobacteria exhibited positive correlations with Ca^2^^+^, HCO_3_^−^, and Cl^−^, suggesting strong osmoadaptive traits such as spore formation and tolerance to ionic stress. These bacteria are well known for producing mycelia and spores, which enable them to endure various abiotic stresses. Their ability to thrive in hypersaline environments allows them to play important roles in sustainable agriculture, including promoting nitrogen fixation, mobilizing phosphorus and potassium, and enhancing crop resistance [30,31,32]. The observed increase in Actinobacteria abundance with rising Ca^2^^+^ concentrations highlights their adaptation to salt-based ionic conditions, consistent with previous findings on the association between Actinobacteria and calcium salts [33]. Chloroflexi generally showed negative correlations with most salinity ions; however, certain genera such as *norank_f_Anaerolineaceae* displayed niche-specific adaptations, highlighting the functional and taxonomic complexity within this phylum (Figure 7). Desulfobacteriota, a group closely linked to sulfur metabolism, plays an important role in mediating microbial responses to salt stress [34]. Its significant correlations with HCO_3_^−^, Cl^−^, and Ca^2^^+^ suggest that imbalances in salinity ion composition may disrupt sulfur metabolic pathways, thereby restricting the colonization and activity of Desulfobacteriota. Similarly, Chloroflexi, a common photosynthetic bacterial group in saline environments [35], shared a similar ion correlation pattern, implying that microbial communities in saline tidal flats may regulate photosynthesis through sulfur-related pathways in response to salt stress.

Notably, Acidobacteriota, often oligotrophic and prevalent in low-nutrient marine sediments [36], peaked early in reclamation and again in late stages under different vegetation covers (Figure 2 and Figure 3). Their dynamic abundance and negative correlation with K^+^ and Cl^−^ suggest that ionic composition modulates their metabolic activity, potentially through inhibition of sulfate reduction pathways [37,38,39], which they frequently mediate. This provides insight into how salinity ion stress may influence broader biogeochemical processes, such as the marine sulfur cycle and microbial stress tolerance mechanisms.

### 4.3. Ecosystem-Level Implications and Study Limitations

These microbial shifts have direct implications for ecosystem health and sustainability. Enhanced bacterial diversity and biomass in rice fields reflect increased microbial functional capacity, supporting higher soil fertility and crop productivity [40]. Conversely, reed beds and bare flats may remain functionally limited due to persistent salinity stress and nutrient deficits [41]. Thus, understanding microbial succession can guide coastal land management strategies, favoring plant–microbe synergy to enhance soil recovery. However, several important limitations of this study should be acknowledged. (1) Rhizosphere soil was not analyzed; only bulk soil samples were considered. Given that microbial communities in the rhizosphere, which directly interact with plant roots, may exhibit different patterns, especially under salt stress, this omission may limit the comprehensiveness of the findings. (2) While reclamation years were standardized, uncontrolled environmental factors such as seasonal variations, hydrological changes, and historical land use could serve as potential confounding variables, affecting the observed results. (3) The functional traits of the microbial communities were inferred from taxonomic data, rather than directly measured. Without the inclusion of metagenomic or transcriptomic analyses, the functional inferences remain speculative and warrant further investigation.

Future studies should incorporate multi-omics approaches to identify key functional groups (e.g., nitrogen fixers, sulfate reducers, osmolyte synthesizers) and assess their activity in situ. Additionally, controlled experiments manipulating specific salinity ions can elucidate causal relationships between ionic composition and microbial community structure. These steps will improve our understanding of microbial resilience and facilitate more targeted coastal restoration efforts.

## 5. Conclusions

This study demonstrates that long-term tidal flat reclamation significantly alters soil physicochemical properties and microbial community dynamics, with clear differentiation among vegetation types. Our results indicate that vegetation configuration plays a critical role in determining soil nutrient enrichment and salinity mitigation. Specifically, rice fields after 50 years of reclamation exhibited the highest enrichment in SOC, TN, and TP, alongside a marked decrease in Cl^−^ and TDS, suggesting greater ecological benefits compared to reed beds and bare flats. Furthermore, we identified HCO_3_^−^, Cl^−^, and K^+^ as dominant drivers of microbial community structure, exceeding TDS and pH in explanatory power. These ions showed strong correlations with key bacterial phyla: HCO3^−^ positively influenced Actinobacteriota, K^+^ supported Proteobacteria likely through osmotic regulation, while Cl^−^ inhibited Acidobacteriota. Our findings also reveal complex niche adaptations at the genus level, including taxa within Chloroflexi that exhibited unexpected halotolerance, diverging from phylum-level patterns.

Based on these insights, we recommend a staged vegetation restoration strategy: (1) establish early reed vegetation within the first 3–5 years to stabilize the soil and initiate salinity reduction; (2) transition to rice cultivation in mid- to late-stage reclamation (>10 years) to promote nutrient accumulation and maximize microbial diversity. Additionally, monitoring specific salinity ions (Cl^−^, K^+^, HCO_3_^−^) and microbial indicators (e.g., Acidobacteriota) is essential for evaluating ecosystem health and guiding adaptive management. These findings contribute to evidence-based planning for sustainable reclamation and biodiversity conservation in coastal wetland ecosystems.

## 6. Future Research Directions

Future studies should further explore the mechanistic links between specific salinity ions and microbial functional traits to better understand how these ions regulate microbial community assembly and ecosystem functioning. Metagenomic and metatranscriptomic approaches can provide deeper insight into the functional adaptations of halotolerant taxa and their contributions to nutrient cycling. In addition, long-term monitoring of reclamation sites with varying vegetation transitions and salinity management strategies is needed to validate the phased restoration model proposed in this study. Expanding the research to include fungal and archaeal communities, as well as their interactions with bacteria, will provide a more holistic understanding of soil microbial ecology under reclamation. Finally, linking microbial community dynamics with ecosystem services such as carbon sequestration and soil fertility could enhance the predictive capacity of reclamation outcomes and inform adaptive coastal management.

## Figures and Tables

**Table 1:** Variations in soil chemical properties across different reclamation years and vegetation types.

Samples	SOC (g kg^−1^)	TN (g kg^−1^)	TP (g kg^−1^)	TK (g kg^−1^)	TDS (g kg^−1^)	pH
**GT_yr1**	2.98 ± 0.07	0.32 ± 0.01	0.51 ± 0.02	12.67 ± 0.15	3.08 ± 0.18	7.85 ± 0.18
**GT_yr3**	3.87 ± 0.24	0.44 ± 0.02	0.72 ± 0.02	14.83 ± 0.21	3.14 ± 0.10	7.74 ± 0.14
**GT_yr5**	3.27 ± 0.22	0.33 ± 0.01	0.57 ± 0.02	13.67 ± 0.15	2.38 ± 0.14	8.17 ± 0.10
**GT_yr10**	2.65 ± 0.09	0.29 ± 0.02	0.53 ± 0.03	14.43 ± 0.60	2.55 ± 0.18	8.38 ± 0.11
**GT_yr50**	11.66 ± 0.27	1.15 ± 0.04	0.77 ± 0.02	13.10 ± 0.62	2.65 ± 0.13	8.15 ± 0.15
**LW_yr1**	4.04 ± 0.09	0.41 ± 0.01	0.56 ± 0.02	14.27 ± 0.25	2.04 ± 0.07	7.54 ± 0.11
**LW_yr3**	11.87 ± 0.09	1.15 ± 0.03	0.63 ± 0.02	15.23 ± 0.25	1.52 ± 0.08	7.96 ± 0.22
**LW_yr5**	13.05 ± 0.52	1.26 ± 0.02	0.74 ± 0.02	14.97 ± 0.47	1.64 ± 0.05	7.71 ± 0.19
**LW_yr10**	13.44 ± 0.09	1.35 ± 0.02	0.91 ± 0.01	15.83 ± 0.12	2.06 ± 0.07	7.55 ± 0.14
**LW_yr50**	13.55 ± 0.09	1.33 ± 0.03	0.80 ± 0.02	15.03 ± 0.59	1.62 ± 0.09	7.59 ± 0.12
**SD_yr10**	3.34 ± 0.09	1.57 ± 0.04	0.65 ± 0.02	16.00 ± 0.26	1.22 ± 0.05	7.98 ± 0.13
**SD_yr50**	20.38 ± 0.27	1.97 ± 0.02	1.04 ± 0.02	12.80 ± 0.56	0.96 ± 0.09	8.00 ± 0.23
**Sources of Variance**	Significance *p*-Value					
	SOC	TN	TP	TK	TDS	pH
**Vegetation**	**0.0008**	**0.0011**	0.0603	**0.0073**	**0.0000**	**0.0003**
**Year**	**0.0087**	**0.0153**	**0.0026**	**0.0015**	**0.0178**	0.5594
**Vegetation × Year**	0.2677	0.1480	0.0972	0.4846	0.4213	**0.0177**

Values are presented as means ± standard deviations (SDs). SOC, soil organic carbon; TN, total nitrogen; TP, total phosphorus; TK, total potassium; TDS, total dissolved salts. Significance (*p*-values) was determined using the Scheirer–Ray–Hare test. Vegetation: vegetation cover types (GT: bare flat; LW: reed bed; SD: rice field); Year: years since reclamation (yr1, yr3, yr5, yr10, yr50). Values in bold indicate *p*-values < 0.05.

## Data Availability

The original contributions presented in this study are included in the article/Supplementary Material. Further inquiries can be directed to the corresponding authors.

## Supplementary Materials

The following supporting information can be downloaded at: https://www.mdpi.com/article/10.3390/microorganisms13061184/s1, Figure S1: Variations in Pearson correlation coefficients among soil K^+^ (a), Na^+^ (b), Ca²^+^ (c), Mg²^+^ (d), Cl^−^l^−^ (e), HCO_3_^−^ (f), SO_4_²^−^ (g), SOC (h), and pH across different vegetation cover types (GT: bare flat, LW: reed bed, SD: rice fiel; Figure S2: Community structure of soil bacterial genera in tidal flats across different reclamation years (1, 3, 5, 10, and 50) and vegetation cover types (a: bare flat, b: reed bed, c: rice field). Genera with a relative abundance of < 0.02 are grouped together as “Others”; Figure S3: Variations in soil organism-level microbiome phenotypes across different vegetation cover types as predicted by BugBase. (a) Kruskal–Wallis H test of phenotypes in bare flat soils across different reclamation years; (b) Kruskal–Wallis H test of phenotypes in reed bed soils across different reclamation years; (c) Wilcoxon rank-sum test of phenotypes in rice field soils between different reclamation years; Table S1: Results of the normality test (Shapiro–Wilk) and homogeneity of variance test; Table S2: Variations in soil salinity ion composition across different reclamation years and vegetation types; Table S3: Redundancy analysis (RDA) ranking the influence of selected environmental variables on soil bacterial communities in tidal flats.
