# Supplementary material for: Vegetation-Driven Changes in Soil Salinity Ions and Microbial Communities Across Tidal Flat Reclamation
Source: Microorganisms. 2025 May 22;13(6):1184. doi: 10.3390/microorganisms13061184 (PMC12195035; doi:10.3390/microorganisms13061184)
Supplement: Supplementary file 1 [file microorganisms-13-01184-s001.zip › microorganisms-3516881-supplementary.pdf]

Vegetation-driven changes in soil salinity ions and microbial communities across tidal flat reclamation

Shumei Cai [1,2,3], Sixin Xu [1,2,3,*], Deshan Zhang [1,2,3], Yun Liang [1,2,3] and Haitao Zhu [1,2,3,*]

[1] Institute of Eco-Environment and Plant Protection, Shanghai Academy of Agricultural Sciences, Shanghai 201403, China; caishumei@saas.sh.cn (S.C.)
[2] Key Laboratory of Low-Carbon Green Agriculture, Ministry of Agriculture and Rural Affairs, Shanghai 201403, China
[3] Shanghai Key Laboratory of Horticultural Technology, Shanghai 201403, China
* Correspondence: xsxofsaas@163.com (S.X.); htzhu123@163.com (H.Z.)

Table S1. Results of the normality test (Shapiro–Wilk) and homogeneity of variance test (Bartlett's test).

Variables	*p*-Value of the Normality Test	*p*-Value of the Homogeneity Test
**SOC**	0.0004	0.3669
**TN**	0.0003	0.0041
**TP**	0.0180	0.9490
**TK**	0.1245	0.3216
**pH**	0.4866	0.7307
**TDS**	0.0000	0.4838
**K^+**	0.0065	0.4343
**Na^+**	0.0182	0.0575
**Ca^{2+}**	0.0466	0.0290
**Mg^{2+}**	0.0002	0.0000
**Cl^-**	0.0565	0.0001
**SO_4^{2-}**	0.0003	0.0001
**HCO_3^-**	0.0070	0.2669

Table S2 Variations in soil salinity ion composition across different reclamation years and vegetation types.

**Samples**	**K^+** (g kg^{-1})	**Na^+** (g kg^{-1})	**Ca^{2+}** (g kg^{-1})	**Mg^{2+}** (g kg^{-1})	**Cl^-** (g kg^{-1})	**SO_4^{2-}** (g kg^{-1})	**HCO_3^-** (g kg^{-1})
GT_yr1	0.025±0.003	0.244±0.003	0.479±0.007	0.110±0.001	0.542±0.014	0.402±0.008	0.399±0.010
GT_yr3	0.025±0.003	0.280±0.003	0.433±0.012	0.123±0.002	0.481±0.008	0.395±0.009	0.396±0.011
GT _yr5	0.015±0.001	0.180±0.004	0.362±0.007	0.081±0.003	0.362±0.012	0.448±0.005	0.233±0.004
GT _yr10	0.025±0.001	0.208±0.001	0.333±0.001	0.102±0.001	0.362±0.012	0.385±0.007	0.269±0.005
GT _yr50	0.023±0.001	0.203±0.007	0.380±0.009	0.110±0.001	0.403±0.005	0.426±0.005	0.357±0.014
LW_yr1	0.018±0.001	0.220±0.011	0.244±0.010	0.042±0.001	0.368±0.042	0.261±0.004	0.292±0.009
LW _yr3	0.015±0.002	0.206±0.012	0.157±0.005	0.014±0.001	0.162±0.004	0.122±0.005	0.359±0.002
LW _yr5	0.012±0.001	0.101±0.003	0.177±0.009	0.035±0.003	0.133±0.005	0.325±0.005	0.219±0.012
LW _yr10	0.038±0.001	0.185±0.008	0.317±0.007	0.105±0.001	0.390±0.006	0.365±0.011	0.188±0.006
LW _yr50	0.024±0.002	0.292±0.016	0.215±0.003	0.045±0.007	0.244±0.011	0.379±0.060	0.169±0.011
SD_yr10	0.018±0.002	0.088±0.005	0.183±0.002	0.021±0.001	0.190±0.002	0.166±0.003	0.146±0.006
SD_yr50	0.011±0.001	0.060±0.006	0.116±0.009	0.014±0.003	0.086±0.020	0.083±0.012	0.186±0.013

Sources of Variance	**Significance *p*-Value**						
	K^+	Na^+	Ca^{2+}	Mg^{2+}	Cl^-	SO_4^{2-}	HCO_3^-
Vegetation	**0.0008**	**0.0016**	**0.0000**	**0.0000**	**0.0001**	**0.0000**	**0.0042**
Year	**0.0010**	**0.0205**	0.6150	0.5550	0.0760	0.4377	**0.0131**
Vegetation×Year	0.2978	0.2083	0.4696	0.1361	0.2432	0.5569	0.6218

Values are expressed as mean ± standard deviation (SD). SOC: soil organic carbon; TN: total nitrogen; TP: total phosphorus; TK: total potassium; TDS: total dissolved salts. p-values for significance are obtained from the Scheirer–Ray–Hare test. Vegetation: vegetation cover types (GT: bare flat; LW: reed bed; SD: rice field); Year: years since reclamation (yr1, yr3, yr5, yr10, yr50). Values in bold indicate p-values < 0.05.

Table S3 Redundancy analysis (RDA) ranking the influence of selected environmental variables on soil bacterial communities in tidal flats.

**Environmental Factors**	**RDA1**	**RDA2**	r^2	***p* value**	**Environmental Factors**	**RDA1**	**RDA2**	r^2	***P* value**
K^+	–0.2961	–0.9552	0.4134	**0.002**	SOC	–0.9420	0.3355	0.2188	**0.026**
Na^+	0.6975	–0.7166	0.2565	**0.012**	TN	–0.9465	0.3228	0.1915	**0.040**
Ca^{2+}	0.8842	–0.4672	0.3485	**0.002**	TP	–0.9402	–0.3406	0.2433	**0.009**
Mg^{2+}	0.6444	–0.7647	0.3360	**0.005**	TK	–0.4189	–0.9081	0.1058	0.163
Cl^-	0.7465	–0.6654	0.4249	**0.001**	pH	0.5709	0.8210	0.1058	0.143
SO_4^{2-}	0.2106	–0.9776	0.1563	0.061	TDS	–0.3902	–0.9207	0.3392	**0.001**
HCO_3^-	0.9844	0.1757	0.6146	**0.001**					

RDA1 and RDA2 represent the degree of correlation between environmental factors and the first and second RDA axes, respectively. The r^2 value indicates the coefficient of determination, reflecting the strength of the association between environmental factors and species distribution. A higher r^2 value suggests a stronger influence. The *p*-value tests the significance of the correlation, with $p < 0.05$ indicating a statistically significant relationship between the environmental factor and species distribution. Values in bold indicate *p*-values < 0.05.

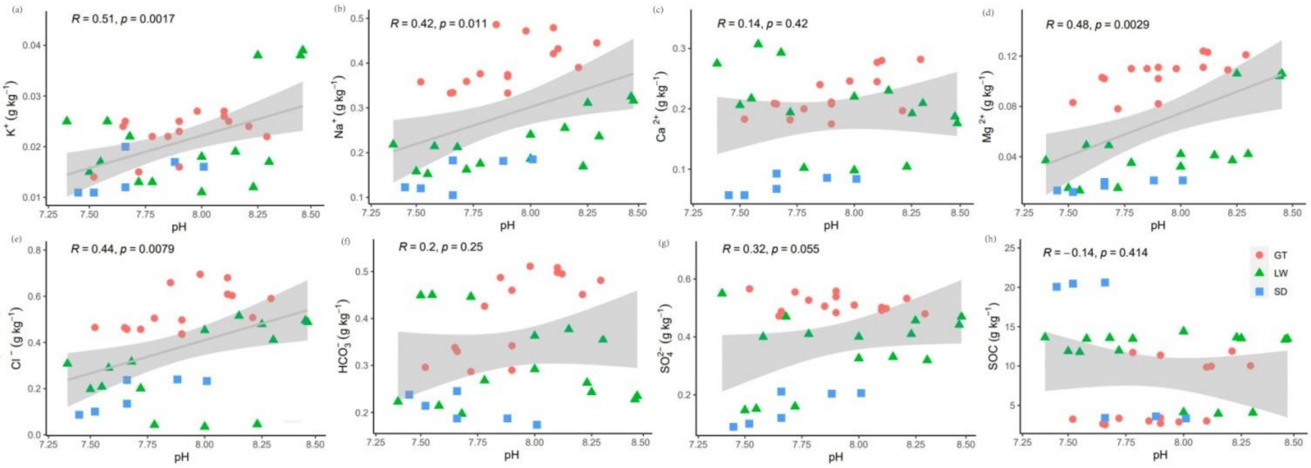

Figure S1. Variations in Pearson correlation coefficients among soil K^+ (**a**), Na^+ (**b**), Ca^{2+} (**c**), Mg^{2+} (**d**), Cl^- (**e**), HCO_3^- (**f**), SO_4^{2-} (**g**), SOC (**h**), and pH across different vegetation cover types (GT: bare flat, LW: reed bed, SD: rice field).

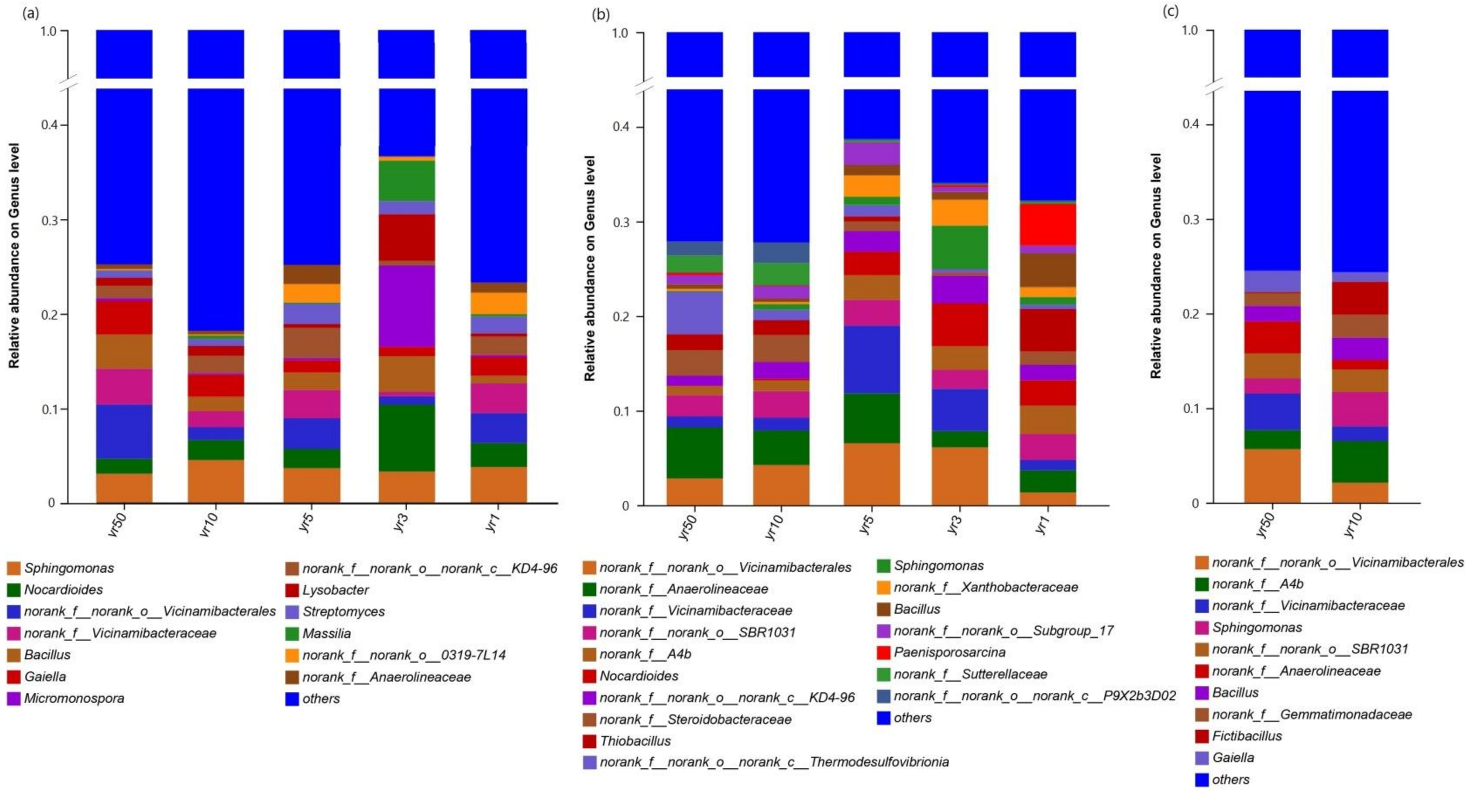

Figure S2. Community structure of soil bacterial genera in tidal flats across different reclamation years (1, 3, 5, 10, and 50) and vegetation cover types (**a**: bare flat, **b**: reed bed, **c**: rice field). Genera with a relative abundance of < 0.02 are grouped together as “Others”.

=

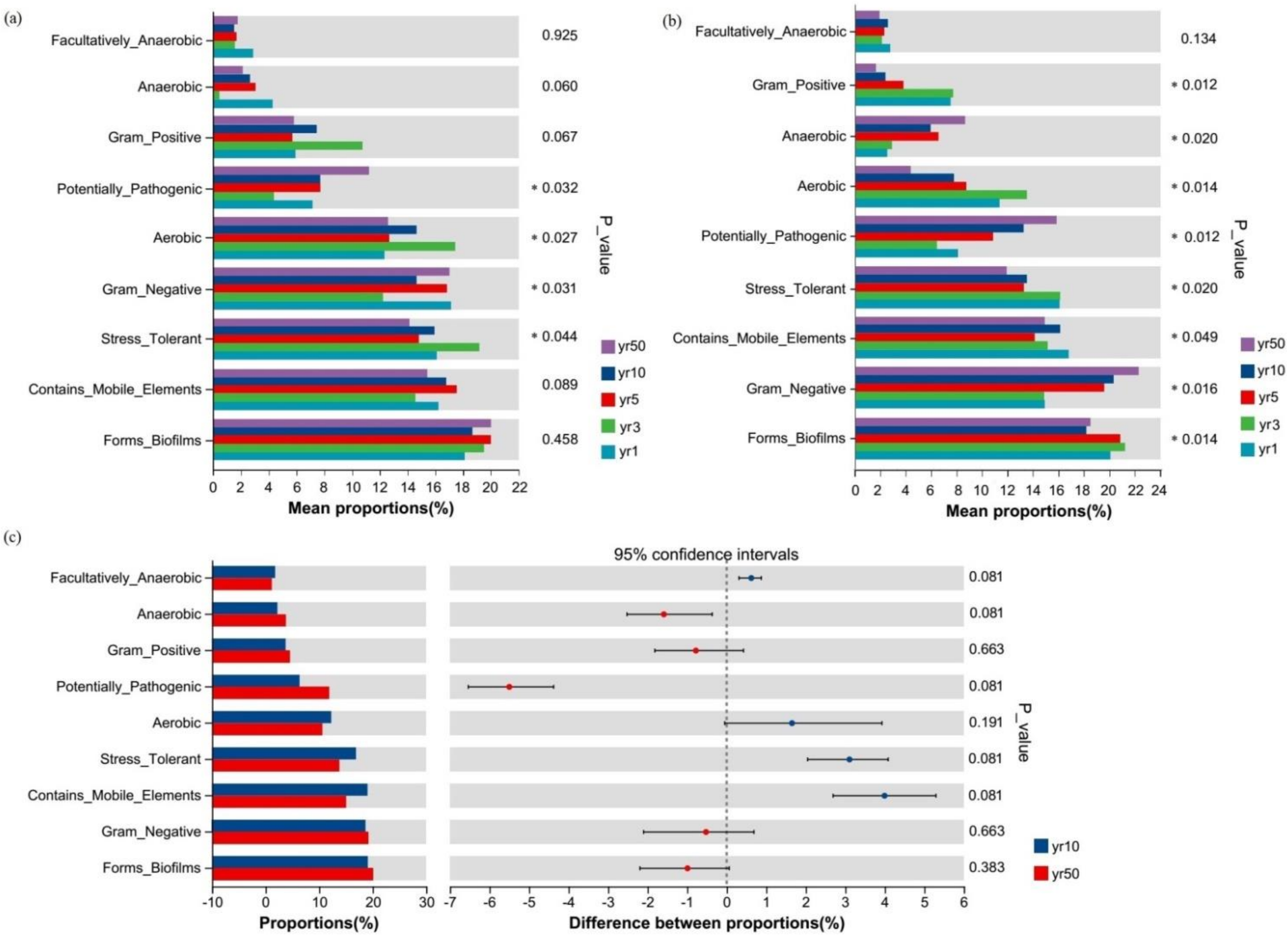

Figure S3. Variations in soil organism-level microbiome phenotypes across different vegetation cover types as predicted by BugBase. (**a**) Kruskal–Wallis H test of phenotypes in bare flat soils across different reclamation years; (**b**) Kruskal–Wallis H test of phenotypes in reed bed soils across different reclamation years; (**c**) Wilcoxon rank-sum test of phenotypes in rice field soils between different reclamation years. The X-axis represents phenotype names, and the Y-axis shows the relative abundance (%) of each phenotype in the samples. Different colors represent different groups. The far-right column displays *p*-values. * indicates $0.01 < p < 0.05$.
